# Study of n-Channel 4H-SiC MOSFETs with a High-*k*/SiO_2_ Stacked Gate Dielectric and a p^+^-Polysilicon Gate

**DOI:** 10.3390/mi17091017

**Published:** 2026-08-27

**Authors:** Zhi Lin, Nuo Cheng, Weicheng Cai, Jianyu Hu, Shengdong Hu

**Affiliations:** School of Microelectronics and Communication Engineering, Chongqing University, Chongqing 400044, China

**Keywords:** 4H-SiC MOSFETs, high-*k* dielectric, p^+^-polysilicon, threshold voltage

## Abstract

In this study, n-channel 4H-SiC MOSFETs with a high-*k*/SiO_2_ stacked gate dielectric and a p^+^-polysilicon gate are proposed and investigated via numerical simulations. The high-*k* dielectric increases the gate capacitance, thereby reducing the specific on-state resistance of the devices. The p^+^-polysilicon increases the work-function difference between the gate and the surface p-SiC, which compensates for the threshold voltage reduction caused by the high-*k* dielectric. Simulation results demonstrate that replacing the SiO_2_ and n^+^-polysilicon with an Al_2_O_3_/SiO_2_ stack and p^+^-polysilicon, respectively, reduces the specific on-resistance by 26.9% while lowering the threshold voltage by only 0.02 V at 25 °C. At 175 °C, the threshold voltage increases by 0.28 V. The temperature coefficient of the threshold voltage is reduced from −6.2 mV/°C to −4.1 mV/°C. In addition, the breakdown voltage, the high-frequency figure of merit, and the switching loss also improve, though the inter-electrode capacitances and the gate-to-drain charge increase. This work provides a practical solution for n-channel 4H-SiC MOSFETs adopting high-*k* gate dielectrics.

## 1. Introduction

Silicon carbide (SiC) is a superior wide-bandgap semiconductor material featuring a high critical electric field strength at breakdown, a low intrinsic carrier density and a high thermal conductivity, making SiC devices suitable for high-voltage and high-temperature operations [1,2,3,4,5]. The 4H-SiC Metal–Oxide–Semiconductor Field-Effect Transistor (MOSFET) is one of the typical SiC power devices. Benefiting from the high critical electric field strength of SiC, the area of a 4H-SiC MOSFET chip is much smaller than that of its silicon counterpart. Parasitic junction capacitors of 4H-SiC MOSFETs are also smaller, leading to shorter switching times, lower switching losses, and higher switching frequency. High-frequency switching reduces the value, along with the size, of the energy storage components (such as capacitors and inductors) within the circuit. Therefore, power converters based on 4H-SiC MOSFETs are compact and efficient. Thus, 4H-SiC MOSFETs are gradually replacing high-voltage silicon devices and are expanding into fields such as data centers, electric vehicles, renewable energy systems, industrial power supplies and motor drives [6,7]. At present, the performance of 4H-SiC MOSFETs remains far below the material’s theoretical limit. One contributing factor lies in the low channel electron mobility, which causes the channel resistance to be a large proportion of the total on-resistance [4,8]. Apart from improving the channel electron mobility through the process technique [9], the improvement methods in the device structure include reducing the channel length [10,11], shrinking the cell pitch [12,13], and introducing the trench gate [14,15,16]. Another approach is to replace the silicon oxide (SiO_2_) gate dielectric with high-*k* gate dielectrics [17,18,19,20,21], such as Al_2_O_3_, HfO_2_, etc. Previous studies have demonstrated that high-*k* gate dielectrics can reduce the equivalent oxide thickness and decrease the channel resistance [22,23,24,25]. However, the threshold voltage (*V*_TH_) of the device decreases as well, increasing the risk of false turn-on [26,27,28].

To address the threshold voltage degradation caused by high-*k* dielectrics in 4H-SiC MOSFETs, this paper proposes using a p^+^-polysilicon gate on the high-*k* dielectric. Benefiting from the high work function of p^+^-polysilicon, the device maintains a sufficient threshold voltage. The aim of this study is to propose a practical solution for n-channel 4H-SiC MOSFETs using high-*k* gate dielectrics, which reduces the specific on-resistance (*R*_ON,SP_) while suppressing threshold voltage degradation. The main innovation lies in combining a high-*k*/SiO_2_ stacked gate dielectric with a p^+^-polysilicon gate in SiC MOSFETs. The proposed device features a 1200 V target voltage class, an operating temperature range of −55 °C to 175 °C, and a maximum gate driving range of −10 V to 22 V. The expected advantages of the proposed device over conventional SiC MOSFETs are its lower specific on-resistance and reasonable threshold voltage. The following sections explain the device’s mechanism, demonstrate the effect of the p^+^-polysilicon gate using Technology Computer-Aided Design (TCAD) simulations, and analyze the influence of the dielectric constant. The main conclusion of this paper will be summarized in the last section.

## 2. Concepts and Methods

**Figure 1:**
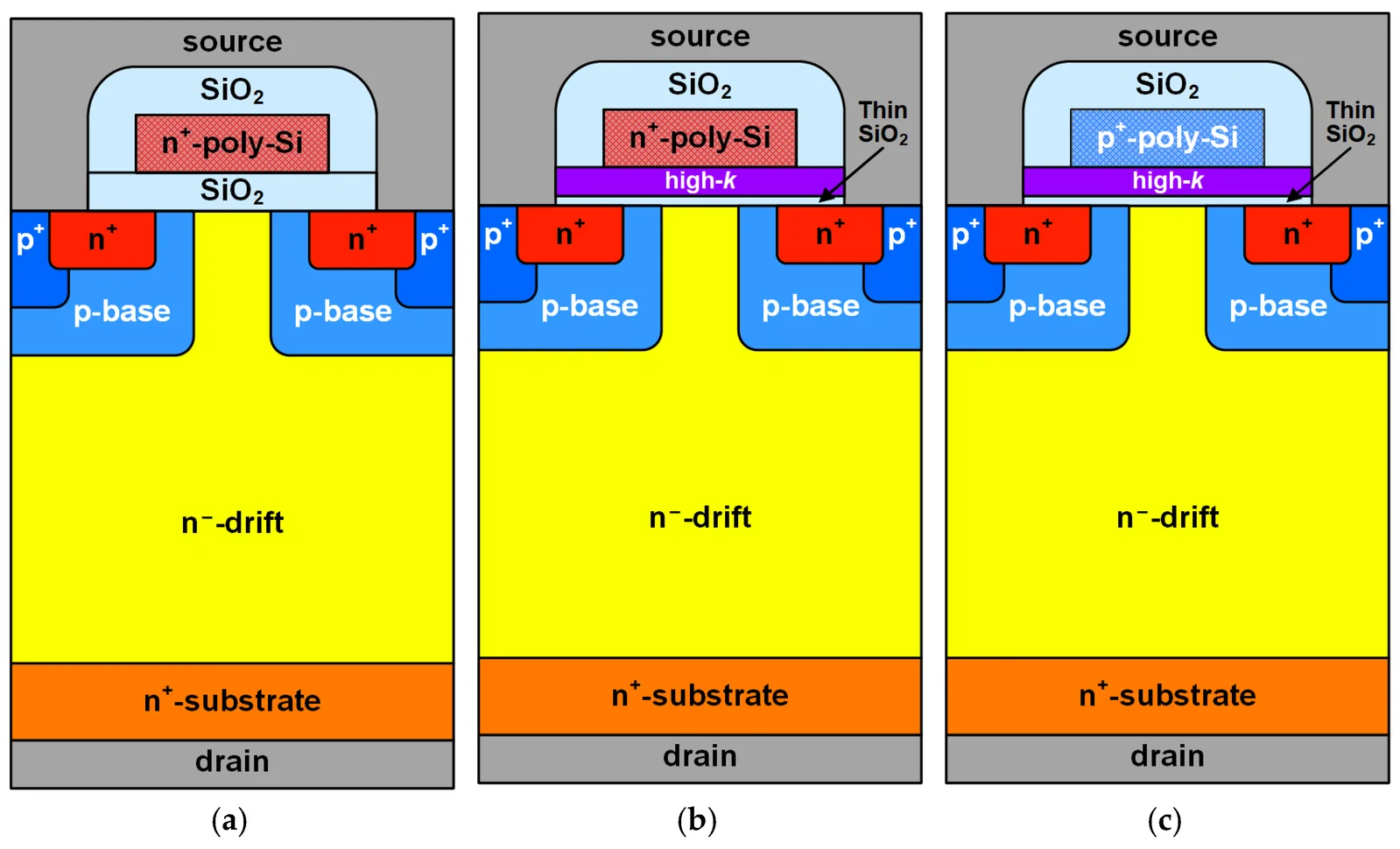
Schematic cross-sections of n-channel 4H-SiC MOSFETs: (**a**) conventional n^+^-poly/SiO_2_ MOSFET, (**b**) conventional n^+^-poly/high-*k* MOSFET, and (**c**) proposed p^+^-poly/high-*k* MOSFET.

Figure 1 shows schematic cross-sections of the planar n-channel 4H-SiC MOSFETs under study. Figure 1a shows a conventional SiC MOSFET with a SiO_2_ gate dielectric and an n^+^-polysilicon gate (n^+^-poly/SiO_2_ MOSFET). Figure 1b shows a conventional SiC MOSFET with a high-*k*/SiO_2_ stacked gate dielectric and an n^+^-polysilicon gate (n^+^-poly/high-*k* MOSFET). Figure 1c shows the proposed SiC MOSFET with a high-*k*/SiO_2_ stacked gate dielectric and a p^+^-polysilicon gate (p^+^-poly/high-*k* MOSFET). A thin SiO_2_ layer is sandwiched between the high-*k* layer and the SiC region because it has a good interface with silicon carbide and a large bandgap. The manufacturability of the p^+^-polysilicon/high-*k* stack has been proven by previous works [29], and all parameters except for the gate and gate dielectric materials are kept identical.

The threshold voltage of a MOS structure is as follows [1]:

(1) $$V_{TH}=\left(\Phi_{GS}-\frac{Q_{OX}}{C_{OX}}\right)+2\Psi_{FB}+\frac{\sqrt{4\varepsilon_{SiC}qN_{A}\Psi_{FB}}}{C_{OX}}$$

where $\Phi_{GS}$ is the work-function difference between the gate and the surface p-SiC, $C_{OX}$ is the oxide capacitance, $Q_{OX}$ is the fixed oxide charge, $\Psi_{FB}$ is the Fermi potential of the p-SiC, $N_{A}$ is the doping concentration in the channel, $\varepsilon_{SiC}$ is the dielectric coefficient of SiC, and q is the elementary charge. $\Psi_{FB}$ can be calculated as

(2) $$\Psi_{FB}=\frac{kT}{q}\ln\left(\frac{N_{A}}{n_{i}}\right)$$

where k is the Boltzmann constant, T is the temperature, and $n_{i}$ is the intrinsic carrier concentration of SiC. When a high-*k*/SiO_2_ stacked gate dielectric is used, $C_{OX}$ can be calculated as

(3) $$C_{OX}=\frac{\varepsilon_{0}}{\frac{t_{HK}}{\varepsilon_{HK}}+\frac{t_{{SiO}_{2}}}{\varepsilon_{{SiO}_{2}}}}$$

where $\varepsilon_{HK}$ and $\varepsilon_{{SiO}_{2}}$ are, respectively, the dielectric constant of the high-*k* dielectric and SiO_2_; $t_{HK}$ and $t_{{SiO}_{2}}$ are, respectively, the thickness of the high-*k* layer and the thin SiO_2_ layer; and $\varepsilon_{0}$ is the permittivity in vacuum. Since $\varepsilon_{HK}>\varepsilon_{{SiO}_{2}}$, the gate capacitance of the high-*k*/SiO_2_ stack is larger than that of the pure SiO_2_ with the same thickness. Therefore, the n^+^-poly/high-*k* MOSFET has a lower threshold voltage than the n^+^-poly/SiO_2_ MOSFET. The larger the dielectric constant, the lower the threshold voltage. A low-threshold voltage is undesirable, as it increases the risk of false turn-on of the device. There are several high-*k* materials available [17], such as Al_2_O_3_, Y_2_O_3_ and HfO_2_. The key physical parameters of the dielectrics used in this study are listed in Table 1.

**Figure 2:**
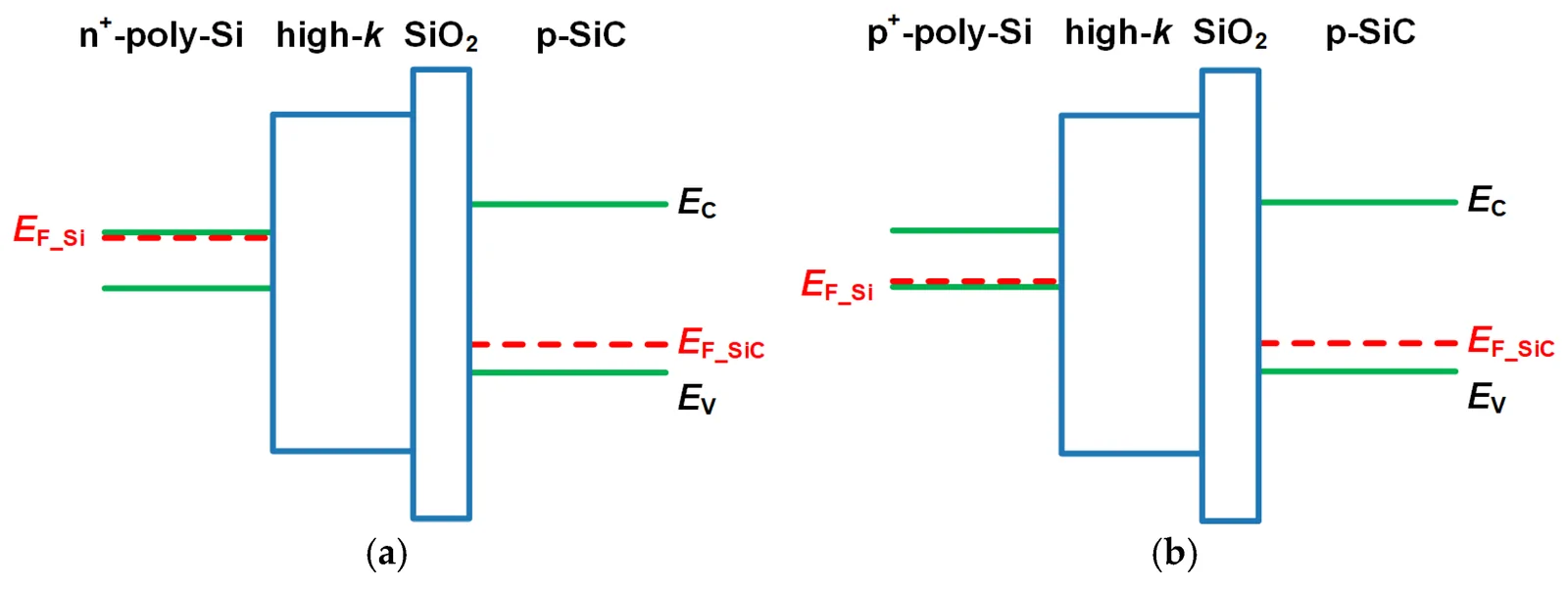
Band alignments of polysilicon/high-*k* dielectric/SiO_2_/p-SiC structures: (**a**) n^+^-polysilicon and (**b**) p^+^-polysilicon.

The effect of the p^+^-polysilicon gate on the threshold voltage can be explained using band alignments of polysilicon/high-*k* dielectric/SiO_2_/p-SiC structures, as shown in Figure 2. 

The work-function difference in (1) can be calculated as [30]

(4) $$\Phi_{GS}=\frac{E_{F\_Si}-E_{F\_SiC}}{q}$$

where $E_{F\_Si}$ and $E_{F\_SiC}$ are the Fermi energy levels of the polysilicon and the p-SiC, respectively. Since the work function of p^+^-polysilicon usually exceeds that of n^+^-polysilicon, its Fermi energy level is lower. As a result, $\Phi_{GS}$ between the p^+^-polysilicon and p-SiC is larger than that between the n^+^-polysilicon and p-SiC. The difference depends on the doping concentration of polysilicon regions, as discussed later. This can compensate for the threshold voltage reduction induced by the high-*k* gate dielectric and mitigate the risk of false turn-on of the device.

**Figure 3:**
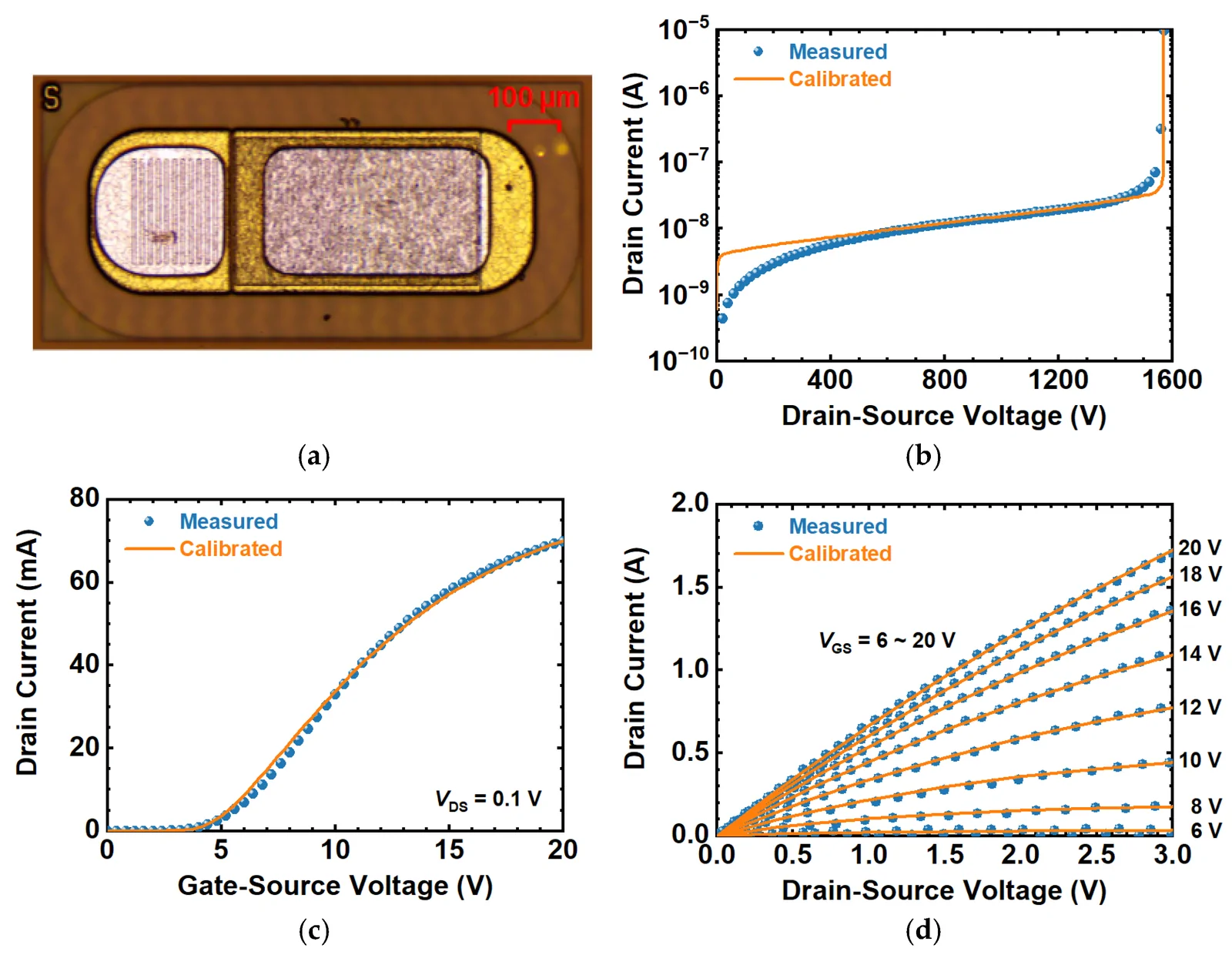
(**a**) Micrograph of the fabricated n^+^-poly/SiO_2_ SiC MOSFET. Comparison of calibrated simulation results with experimental data: (**b**) forward blocking characteristics, (**c**) transfer characteristics, and (**d**) output characteristics. Simulation models were calibrated based on experimental data.

The conventional n^+^-poly/SiO_2_ MOSFET, designed and fabricated on a commercial 6-inch platform, was used to calibrate the physical models adopted in TCAD simulations. Its cell pitch and channel length are 4.8 μm and 0.5 μm, respectively. An 11 μm thick n^−^-layer doped with nitrogen serves as its drift region, and the nitrogen concentration is 1 × 10^16^ cm^−3^. Multiple high-temperature aluminum implantations form the retrograde profile of the p-base layer, which has a surface and peak concentration of 3 × 10^16^ cm^−3^ and 3 × 10^18^ cm^−3^, respectively, and a thickness of 1.1 μm. JFET implantations were also used to reduce on-resistance (*R*_ON_). The active area of the fabricated chip is 0.2 mm^2^. Figure 3a depicts the device micrograph. Its electrical performances were characterized on-wafer using a manual probe station and a semiconductor parameter analyzer at room temperature, and the results are shown in Figure 3b–d. The TCAD tool Sentaurus Device [31] was used to optimize and analyze the electrical performance of the devices. The employed mobility models included the DopingDependence, HighFieldSaturation, and Lombardi models, while traps and fixed charges at the SiO_2_–SiC interface were also involved. Generation–recombination processes were described by the Shockley–Read–Hall and Auger recombination models, while the Okuto–Crowell avalanche model was used to analyze the blocking characteristics. The Slotboom model was used to describe the bandgap-narrowing effect. All simulations incorporated Fermi–Dirac statistics, incomplete ionization, and the anisotropic properties of SiC. The simulated and measured static characteristics are compared in Figure 3b–d. The simulation models were calibrated based on experimental data. The calibrated fixed oxide charge density and trap density are 2.3 × 10^12^ cm^−2^ and 4.6 × 10^12^ cm^−2^, respectively. They were used to explore the electrical behaviors of the studied MOSFETs. The key device parameters used in the simulations are listed in Table 2; however, the proposed device itself has not been experimentally validated.

## 3. Results and Discussion

**Figure 4:**
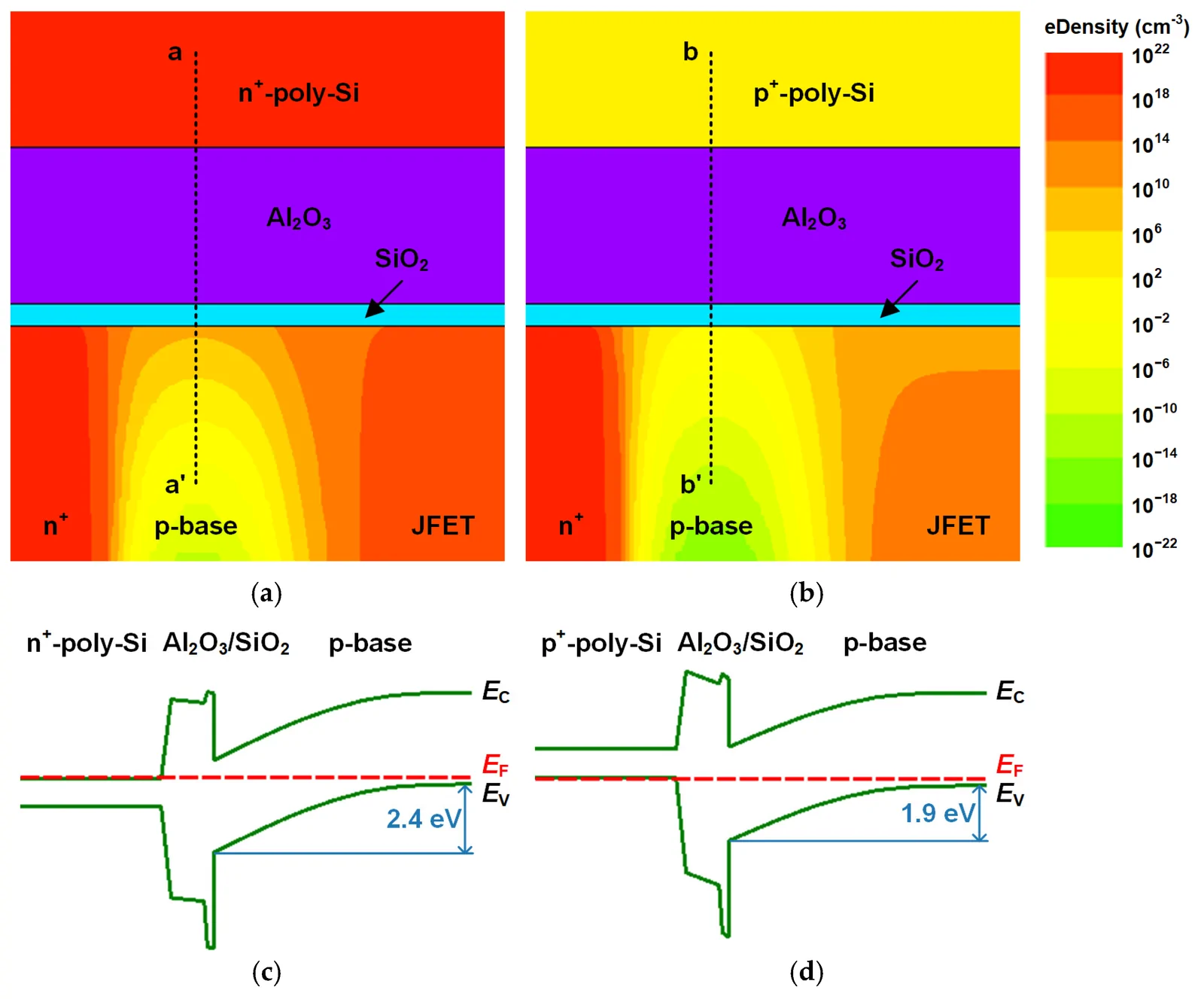
Simulated two-dimensional electron density distributions in and around the channel under zero-bias conditions (*V*_GS_ = 0 V, *V*_DS_ = 0 V) for (**a**) the n^+^-poly/high-*k* MOSFET and (**b**) the p^+^-poly/high-*k* MOSFET. (**c**) Band diagram along the channel center aa′ of the n^+^-poly/high-*k* MOSFET. (**d**) Band diagram along the channel center bb′ of the p^+^-poly/high-*k* MOSFET. Al_2_O_3_ is chosen as the high-*k* dielectric.

Figure 4 shows the simulated two-dimensional electron distribution in and around the channel under zero-bias condition (*V*_GS_ = 0 V, *V*_DS_ = 0 V) for high-*k* MOSFETs with an n^+^-polysilicon gate and a p^+^-polysilicon gate, and the corresponding band diagram along the channel center. Al_2_O_3_ was chosen as the high-*k* dielectric. The electron density in the p^+^-polysilicon region is extremely low. Compared with Figure 4a, the electron density of the channel in Figure 4b is lower, which is caused by the higher work function of p^+^-polysilicon than that of n^+^-polysilicon. Due to the larger work-function difference in the p^+^-poly/high-*k* MOSFET, its band bending (1.9 eV) is smaller than that (2.4 eV) of the n^+^-poly/high-*k* MOSFET. The lower surface potential results in fewer electrons in the channel. Therefore, a higher gate voltage is required to achieve strong inversion of the channel, which means that its threshold voltage is larger.

**Figure 5:**
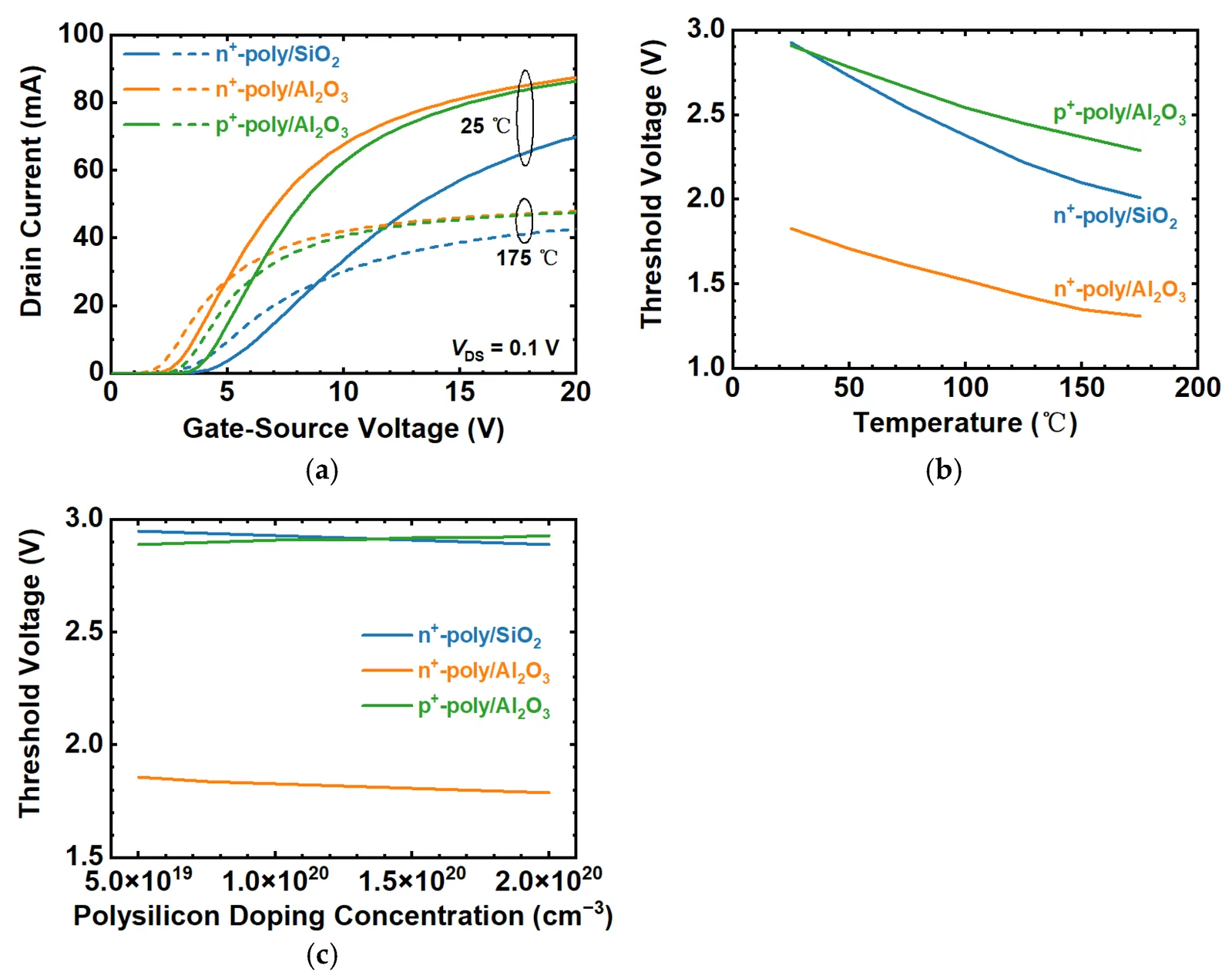
(**a**) Comparison of the transfer characteristics of the three devices at 25 °C and 175 °C. Influence of (**b**) temperature and (**c**) the polysilicon doping concentration on the threshold voltage. The threshold voltage is extracted at *I*_D_ = 0.1 mA, and Al_2_O_3_ is chosen as the high-*k* dielectric.

Figure 5 presents the transfer characteristics of the three devices and their sensitivity to temperature and polysilicon doping concentration when Al_2_O_3_ is chosen as the high-*k* dielectric. The threshold voltage is extracted at *I*_D_ = 0.1 mA. Near room temperature, the threshold voltage of the p^+^-poly/Al_2_O_3_ MOSFET is slightly smaller than that of the n^+^-poly/SiO_2_, but it performs better than the latter at high temperatures. At 25 °C, the threshold voltages of the n^+^-poly/SiO_2_ MOSFET, the n^+^-poly/Al_2_O_3_ MOSFET and the p^+^-poly/Al_2_O_3_ MOSFET are 2.93 V, 1.83 V, and 2.91 V, respectively. At 175 °C, their values are 2.01 V, 1.31 V and 2.29 V, respectively. The differences between the p^+^-poly/Al_2_O_3_ MOSFET and the n^+^-poly/SiO_2_ MOSFET are 0.02 V at 25 °C and −0.28 V at 175 °C. It is clear that high-*k* gate dielectric will reduce the threshold voltage, and the p^+^-polysilicon gate compensates for a portion of the threshold voltage loss. Compared with the n^+^-poly/Al_2_O_3_ MOSFET, the threshold voltage of the p^+^-poly/Al_2_O_3_ MOSFET increases by approximately 1 V. As the gate-source voltage increases, their conducting capabilities become increasingly similar. Furthermore, high-*k* gate dielectric devices exhibit a smaller threshold voltage reduction at high temperatures, as shown in Figure 5b. The threshold voltage temperature coefficients of the n^+^-poly/SiO_2_ MOSFET, the n^+^-poly/Al_2_O_3_ MOSFET and the p^+^-poly/Al_2_O_3_ MOSFET are −6.2 mV/°C, −3.5mV/°C and −4.1 mV/°C, respectively. The threshold voltage decreases as temperature rises because the increasing intrinsic carrier concentration lowers the channel Fermi potential. Moreover, because the $C_{OX}$ value of high-*k* gate MOSFETs is larger than that of the n^+^-poly/SiO_2_ MOSFET, the overall influence of the channel Fermi potential is weakened. Thus, their threshold voltage is less affected by temperature. Figure 5c shows the influence of the polysilicon doping concentration, which ranges from 5 × 10^19^ cm^−3^ to 2 × 10^20^ cm^−3^, on the threshold voltage. Increasing the polysilicon doping concentration lowers the threshold voltage of n^+^-polysilicon devices but raises that of p^+^-polysilicon devices. This is because the work function decreases for the former and increases for the latter. Within the investigated range, the threshold voltage varies by less than 0.1 V. Meanwhile, the threshold voltage difference between the n^+^-poly/Al_2_O_3_ MOSFET and the p^+^-poly/Al_2_O_3_ MOSFET remains at approximately 1 V. A higher polysilicon doping concentration yields a better improvement effect.

**Figure 6:**
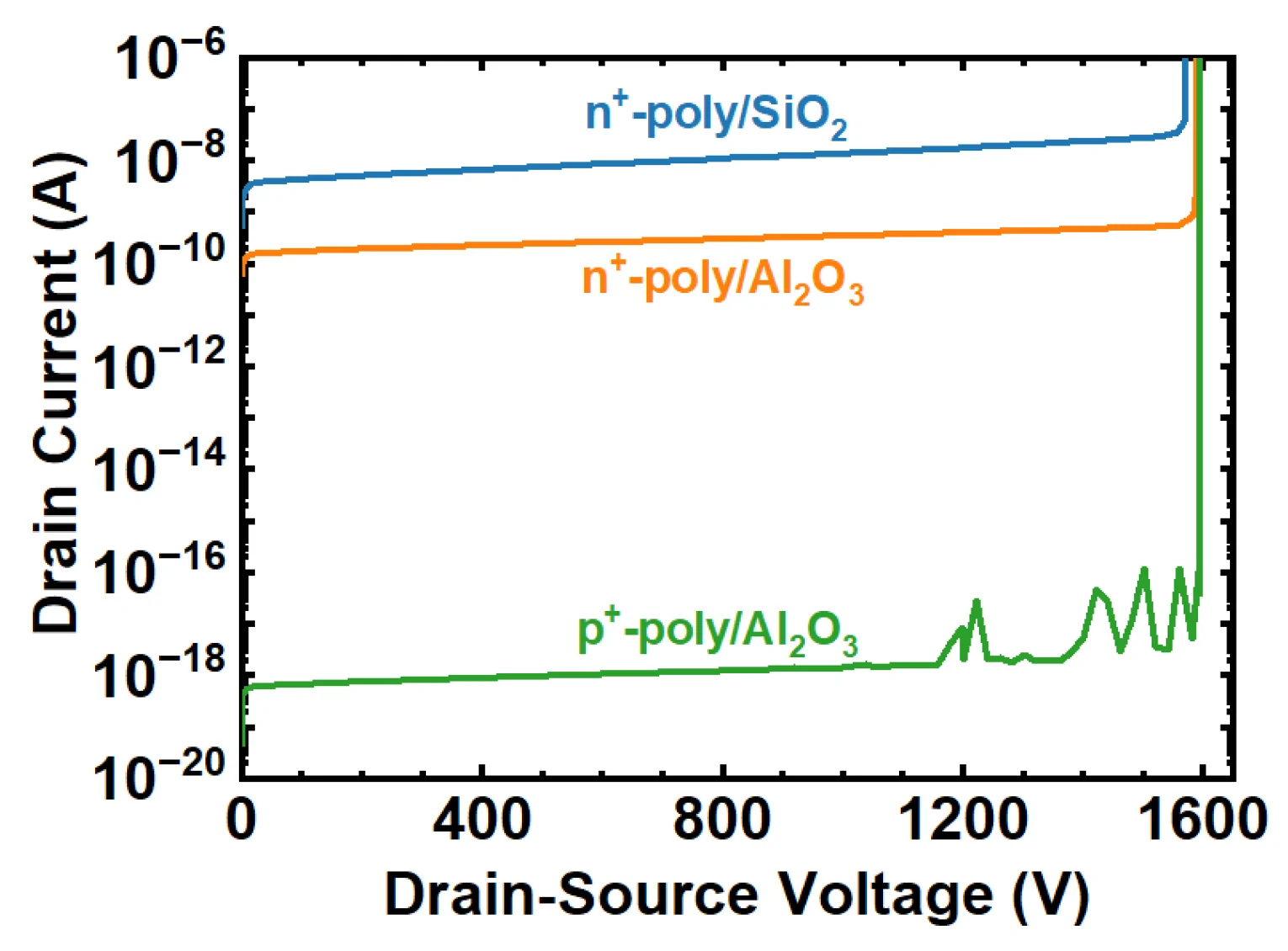
Comparison of forward blocking characteristics. Al_2_O_3_ is chosen as the high-*k* dielectric.

**Figure 7:**
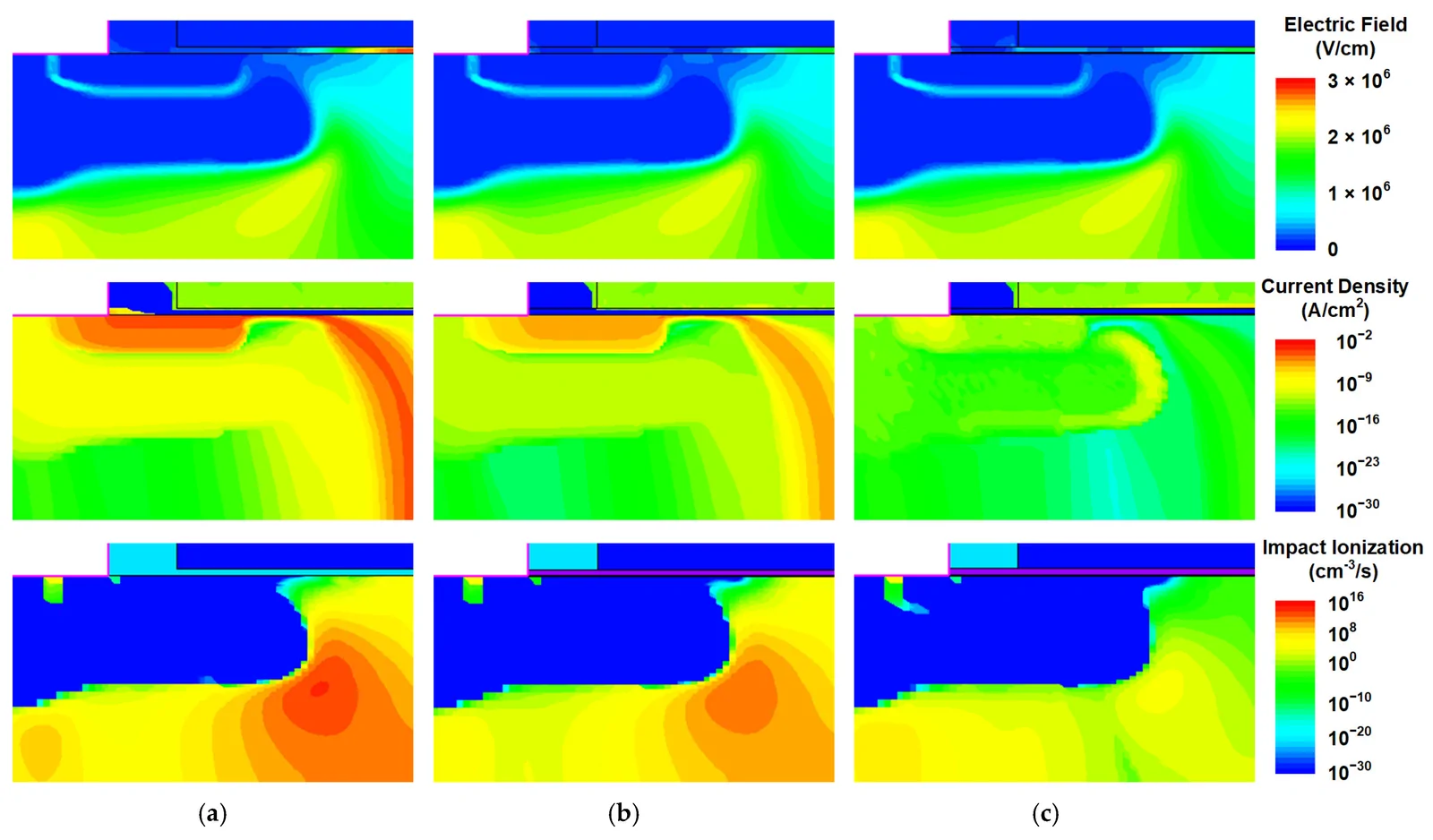
Distributions of electric field, current density and impact ionization rate in (**a**) the conventional n^+^-poly/SiO_2_ MOSFET, (**b**) the conventional n^+^-poly/high-*k* MOSFET, and (**c**) the proposed p^+^-poly/high-*k* MOSFET at 1200 V, respectively. Al_2_O_3_ is chosen as the high-*k* dielectric.

Figure 6 compares the forward blocking characteristics of the three devices when Al_2_O_3_ is chosen as the high-*k* dielectric. The breakdown voltages of the n^+^-poly/SiO_2_ MOSFET, the n^+^-poly/Al_2_O_3_ MOSFET and the p^+^-poly/Al_2_O_3_ MOSFET are 1569 V, 1587 V, and 1593 V, respectively. The distributions of the electric field, current density and impact ionization rate in the three devices at 1200 V are displayed in Figure 7. While there is no significant difference in the distribution of the electric field, the n^+^-poly/SiO_2_ MOSFET has the largest current density, resulting in the strongest avalanche multiplication, largest impact ionization rate, and lowest breakdown voltage. Conversely, the p^+^-poly/Al_2_O_3_ MOSFET has the smallest current density and the largest breakdown voltage. The breakdown voltages of the n^+^-poly/Al_2_O_3_ MOSFET and p^+^-poly/Al_2_O_3_ MOSFET are nearly identical. This is attributed to the relatively low leakage current levels in both devices, even though their leakage currents differ by eight orders of magnitude. The lower leakage current of high-*k* devices is related to the enhanced gate control capability of the high-*k* dielectric. In the off-state, the gate is at zero bias. Positive fixed charges in the dielectric induce negative charges in both the gate electrode and the channel. The channel is in a weak inversion state, as shown in Figure 4, and it contributes to the majority of the leakage current. A large *C*_OX_ of the high-*k* device will increase the amount of negative charges induced on the gate electrode, thereby reducing the amount of negative charges induced in the channel. Therefore, the leakage current decreases and the breakdown voltage increases.

**Figure 8:**
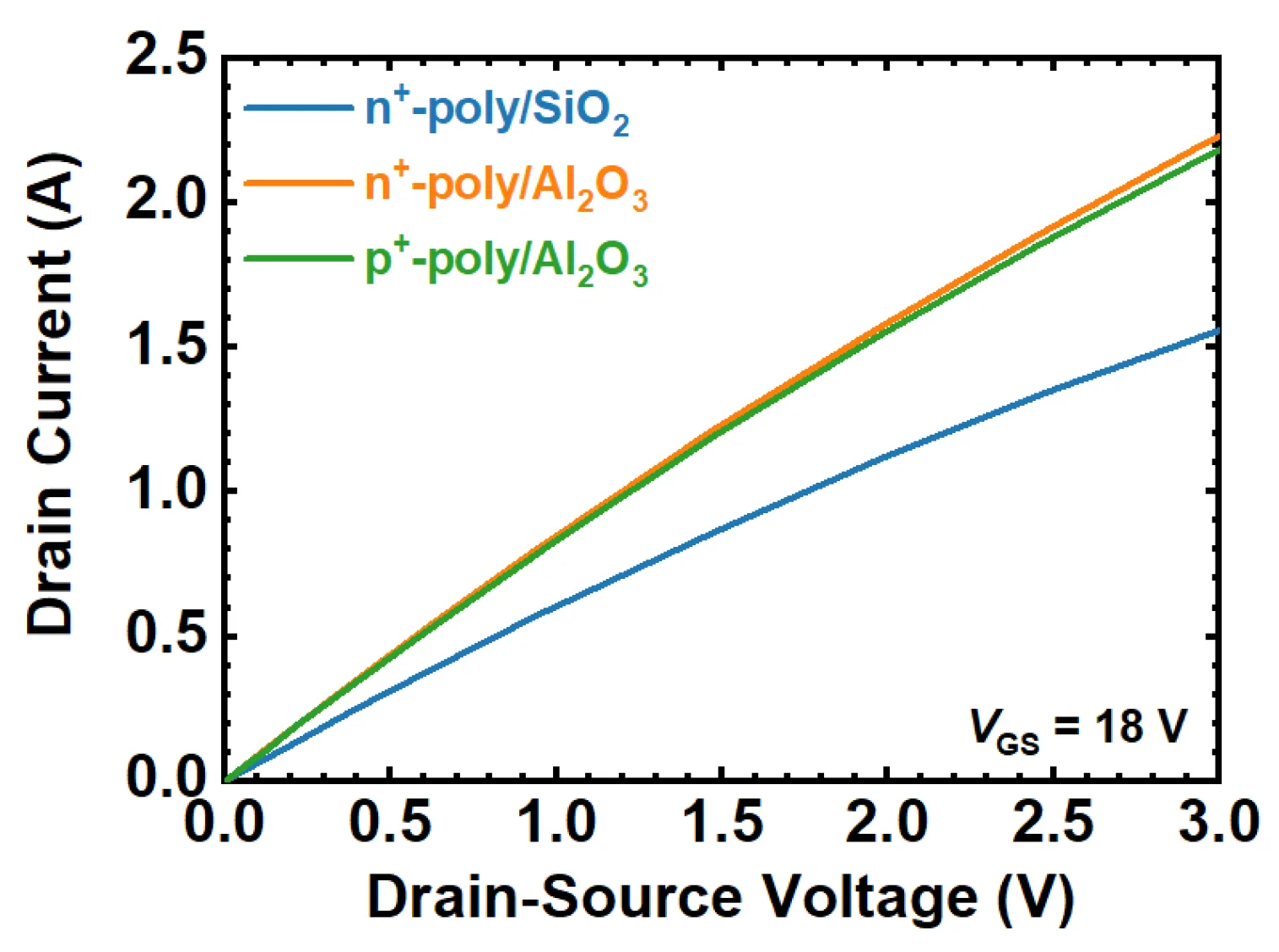
Comparison of output characteristics. Al_2_O_3_ is chosen as the high-*k* dielectric.

Figure 8 compares the output characteristics of the three devices when Al_2_O_3_ is chosen as the high-*k* dielectric. The on-resistances of the n^+^-poly/SiO_2_ MOSFET, the n^+^-poly/Al_2_O_3_ MOSFET and the p^+^-poly/Al_2_O_3_ MOSFET are 1670 mΩ, 1200 mΩ and 1220 mΩ, respectively, extracted under *V*_GS_ = 18 V and *V*_DS_ = 1 V. With an active area of 0.2 mm^2^, the corresponding specific on-resistances are calculated to be 3.34 mΩ·cm^2^, 2.40 mΩ·cm^2^, and 2.44 mΩ·cm^2^, respectively. Both the n^+^-poly/Al_2_O_3_ MOSFET and the p^+^-poly/Al_2_O_3_ MOSFET have lower on-resistance than the n^+^-poly/SiO_2_ MOSFET because the high-*k* gate dielectric induces more channel electrons. The reduction amounts are 28.1% and 26.9%, respectively. Additionally, their conductive abilities at *V*_GS_ = 18 V are comparable, even though their threshold voltages differ by about 1 V. The reason will be explained later. This indicates that the p^+^-polysilicon gate does not sacrifice conductivity of high-*k* gate devices while increasing the threshold voltage.

**Figure 9:**
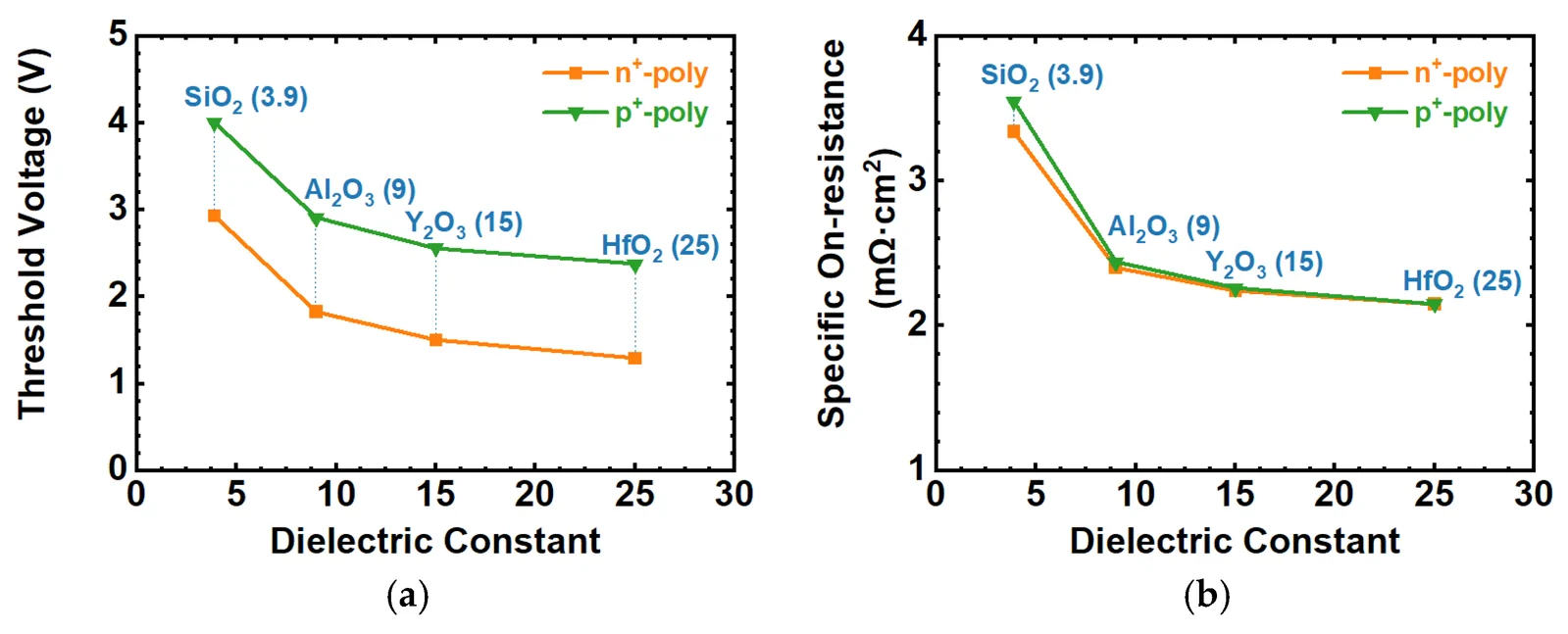
Influence of dielectric coefficient on (**a**) the threshold voltage and (**b**) the specific on-resistance.

Figure 9 shows the influence of dielectric constant on the threshold voltage and the specific on-resistance for materials in Table 1, based on idealized permittivity comparisons. As the dielectric constant increases, $C_{OX}$ increases, causing a reduction in both the threshold voltage and the specific on-resistance, with this influence gradually diminishing when the dielectric constant exceeds 9 (Al_2_O_3_). When the dielectric constant increases from 3.9 (SiO_2_) to 25 (HfO_2_), the threshold voltage of the n^+^-poly/high-*k* MOSFET decreases from 2.93 V to 1.29 V, while that of the p^+^-poly/high-*k* MOSFET decreases from 4.01 V to 2.38 V. For each dielectric material, the difference in threshold voltage between the n^+^-poly/high-*k* MOSFET and the p^+^-poly/high-*k* MOSFET is approximately 1 V. Meanwhile, the specific on-resistance of the n^+^-poly/high-*k* MOSFET decreases from 3.34 mΩ·cm^2^ to 2.15 mΩ·cm^2^, while that of the p^+^-poly/high-*k* MOSFET decreases from 3.55 mΩ·cm^2^ to 2.15 mΩ·cm^2^. As the dielectric constant increases, the difference in specific on-resistance between the n^+^-poly/high-*k* MOSFET and the p^+^-poly/high-*k* MOSFET becomes increasingly smaller. When the dielectric constant exceeds 9 (Al_2_O_3_), the difference becomes almost negligible. The reasons are as follows: The channel electrons induced by the gate include trapped electrons and free electrons, with the gate work function altering the Fermi level, affecting the trapped electron density, and consequently influencing the free electron density. In devices using SiO_2_ as the gate oxide, the channel electron density is comparable to the interface trap density, while the free electron density is also affected by the gate work function, which shows a large difference in specific on-resistance. As the dielectric constant increases, the gate capacitance increases, thereby inducing more channel electrons and causing the fraction of trap electrons to decrease. The effect of the work-function difference becomes smaller and smaller, and the discrepancy in specific on-resistance gradually diminishes.

**Figure 10:**
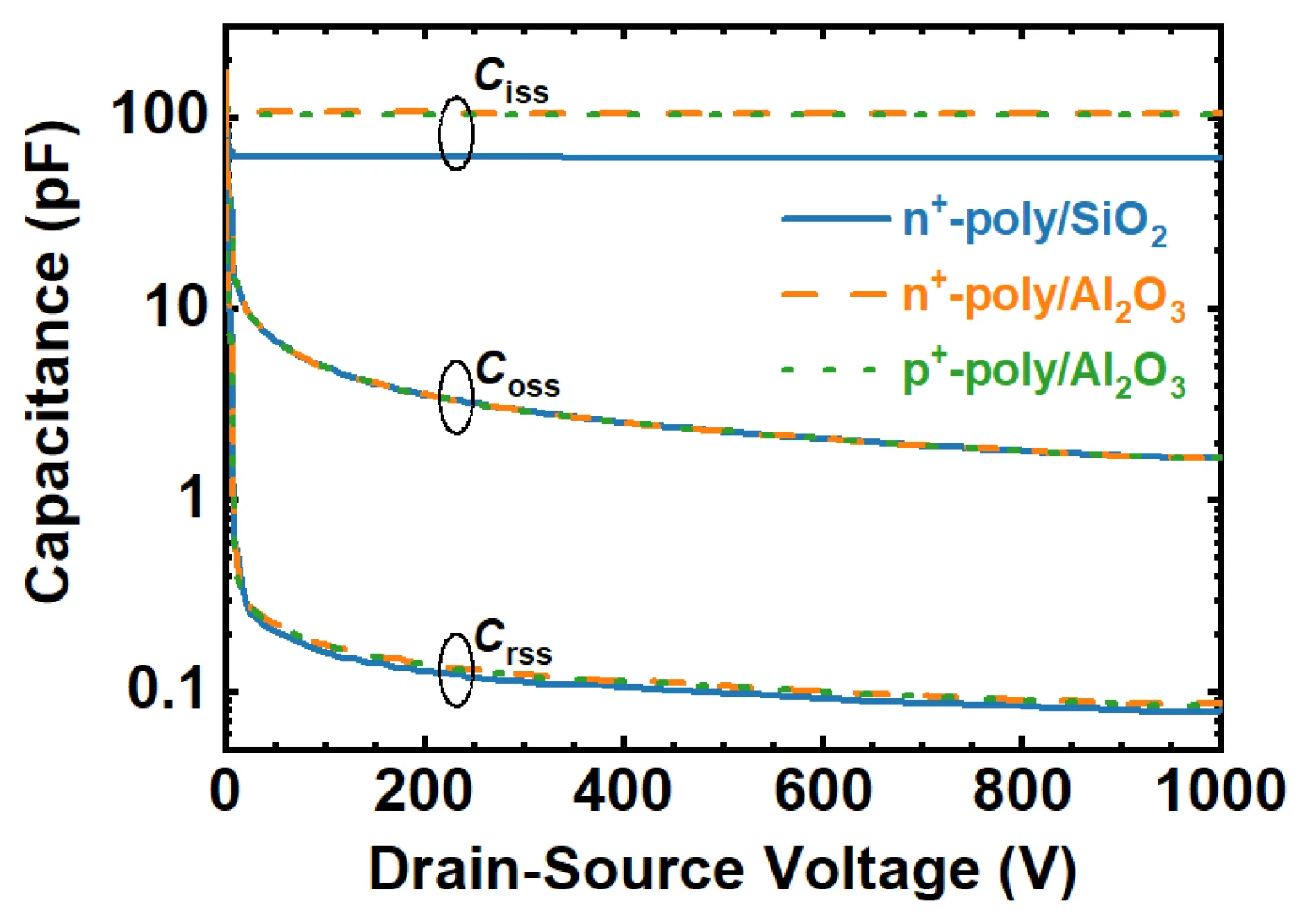
Inter-electrode capacitance comparison of the three devices. Al_2_O_3_ is chosen as the high-*k* dielectric.

**Figure 11:**
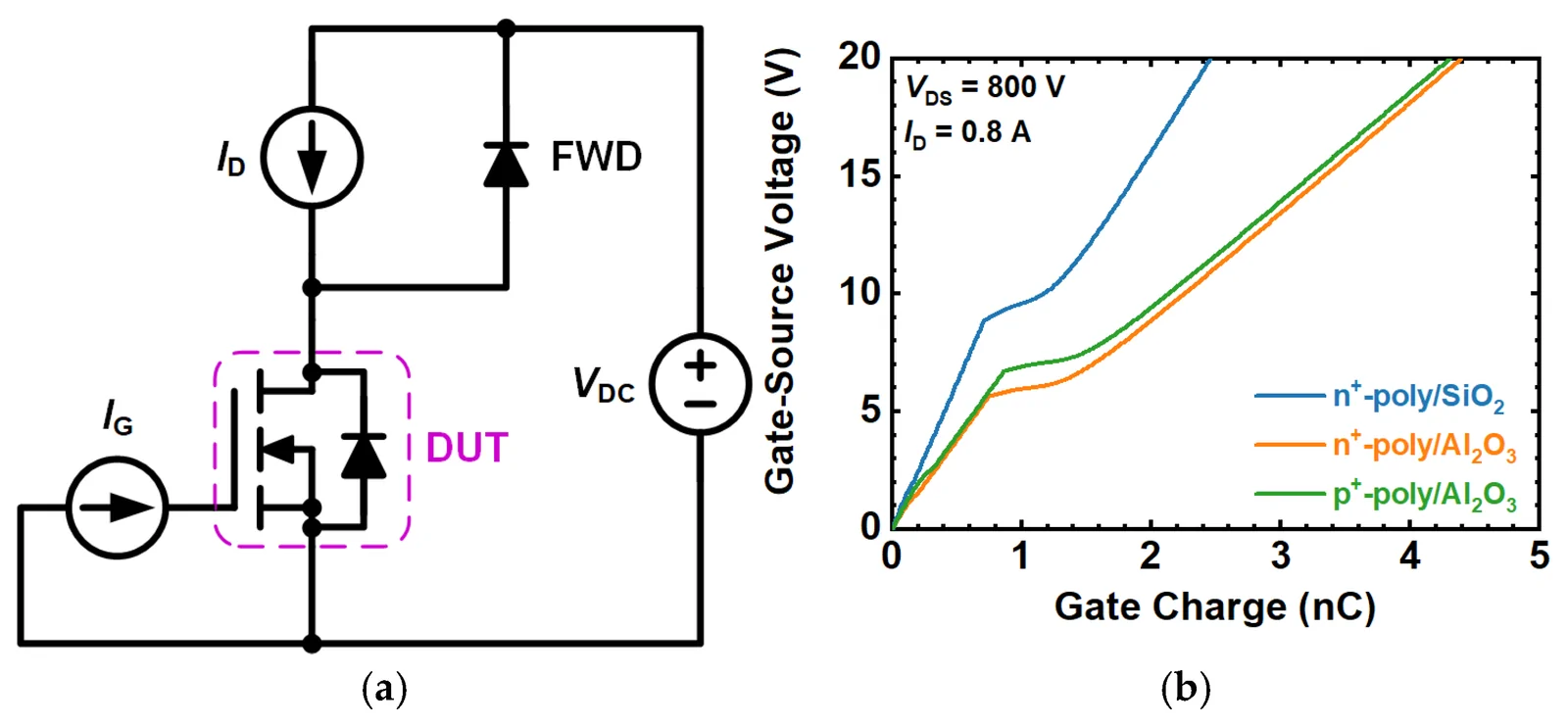
(**a**) Schematic circuit configuration for gate charge extraction. (**b**) Comparison of gate charges. Al_2_O_3_ is chosen as the high-*k* dielectric.

Figure 10 compares the inter-electrode capacitances of the three devices when Al_2_O_3_ is chosen as the high-*k* dielectric. As shown in the figure, the inter-electrode capacitances of the n^+^-poly/Al_2_O_3_ MOSFET and the p^+^-poly/Al_2_O_3_ MOSFET are almost identical. Their input capacitances (*C*_iss_) are larger than that of the n^+^-poly/SiO_2_ MOSFET due to the introduction of a high-*k* gate dielectric. Their reverse transfer capacitances (*C*_rss_ or *C*_GD_) are slightly larger than that of the n^+^-poly/SiO_2_ MOSFET. The output capacitances (*C*_oss_) of the three devices are almost the same. Specifically, the input capacitances of the n^+^-poly/SiO_2_ MOSFET, the n^+^-poly/Al_2_O_3_ MOSFET and the p^+^-poly/Al_2_O_3_ MOSFET at *V*_DS_ = 800 V are 61.7 pF, 95.8 pF and 93.3 pF, respectively. Their output capacitances at *V*_DS_ = 800 V are all 1.8 pF. Their reverse transfer capacitances at *V*_DS_ = 800 V are 84.5 fF, 90.7 fF and 90.2 fF, respectively. The gate-to-drain charge (*Q*_GD_) is extracted using the circuit configuration shown in Figure 11a. The Device Under Test (DUT) is connected to an 800 V power supply (*V*_DC_) through a 0.8 A current source. An ideal FreeWheeling Diode (FWD) is in parallel with the current source. The gate of the DUT is charged by a current source. The gate-to-drain charges of the n^+^-poly/SiO_2_ MOSFET, the n^+^-poly/Al_2_O_3_ MOSFET and the p^+^-poly/Al_2_O_3_ MOSFET extracted from Figure 11b are 0.42 nC, 0.46 nC and 0.46 nC, respectively. Their high-frequency figure-of-merit values (*R*_ON_∗*Q*_GD_) are 700 mΩ∗nC, 556 mΩ∗nC and 567 mΩ∗nC, respectively.

**Figure 12:**
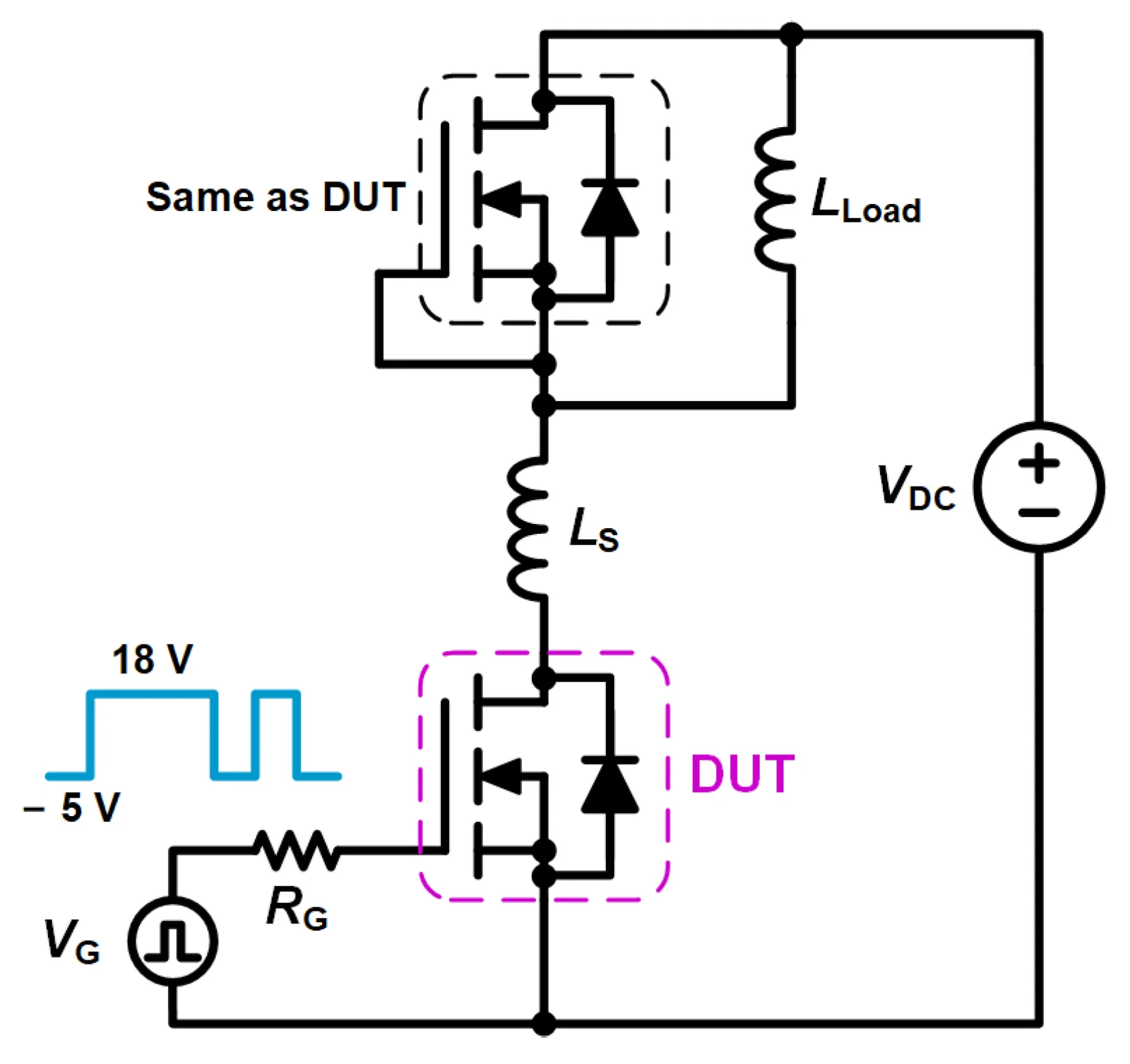
Schematic circuit configuration of the double pulse test.

**Figure 13:**
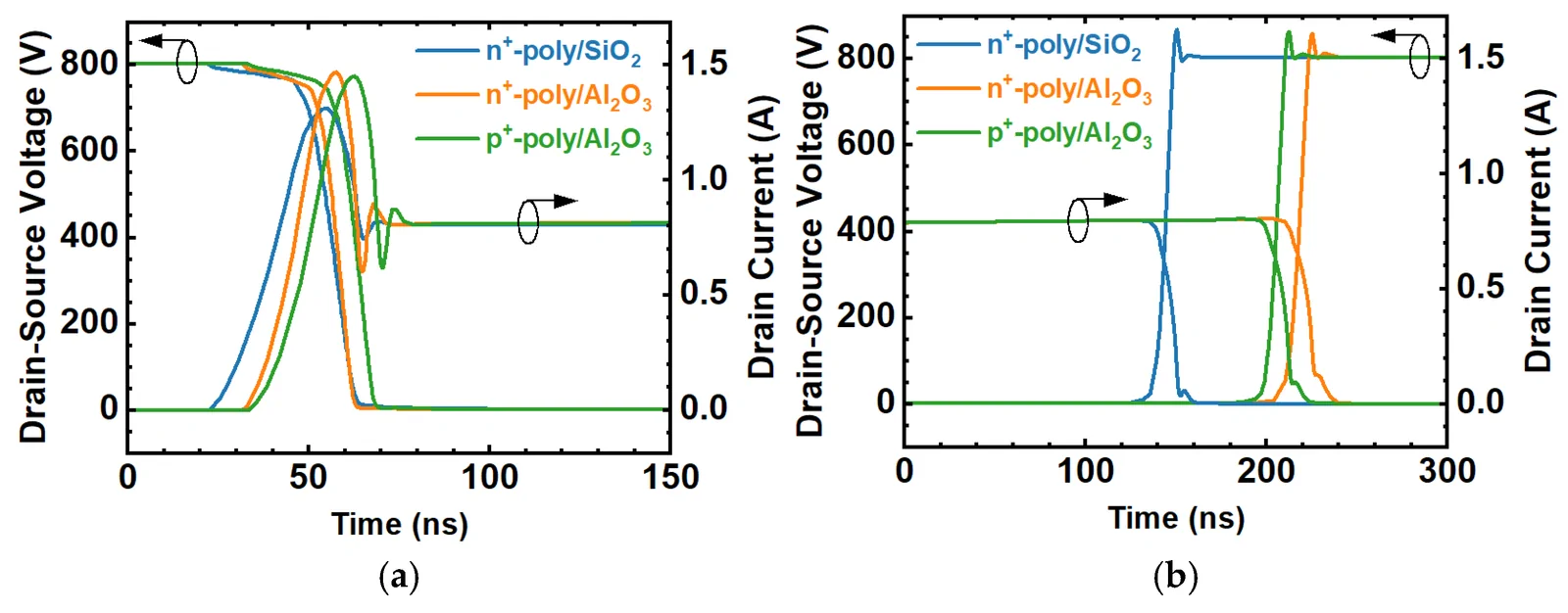
Comparison of switching waveforms of the three devices: (**a**) turn-on waveforms and (**b**) turn-off waveforms. Al_2_O_3_ is chosen as the high-*k* dielectric. The external gate resistor is 500 Ω. The gate drive voltage ranges from −5 V to 18 V.

Figure 12 shows the circuit configuration of the double pulse test used to study the switching characteristics. The gate drive voltage (*V*_G_) ranges from −5 V to 18 V, with an external gate resistor (*R*_G_) of 500 Ω. The load inductor (*L*_Load_) and parasitic inductor (*L*_S_) are 10 mH and 500 nH, respectively. The freewheeling diode is the same as the DUT with its gate shorted to its source. The power supply (*V*_DC_) is 800 V, and the switching current is 0.8 A. Figure 13 shows switching waveforms of the three devices when Al_2_O_3_ is chosen as the high-*k* dielectric. The n^+^-poly/SiO_2_ MOSFET is conducted first and is also turned off first because it has the smallest input capacitance, while the other two devices feature similar input capacitances. However, the threshold voltage of the p^+^-poly/Al_2_O_3_ MOSFET is larger, so it requires a longer charging time and starts conducting later. During the turn-off transient, less time is consumed for its gate to discharge from the drive voltage down to the threshold voltage, so it turns off earlier. The total switching energies of the n^+^-poly/SiO_2_ MOSFET, the n^+^-poly/Al_2_O_3_ MOSFET and the p^+^-poly/Al_2_O_3_ MOSFET are 20.7 µJ, 18.2 µJ and 18.8 µJ, respectively. The p^+^-poly/Al_2_O_3_ MOSFET slightly outperforms the n^+^-poly/SiO_2_ MOSFET.

Table 3 summarizes key electrical parameters of the three devices. Compared with the conventional n^+^-poly/Al_2_O_3_ MOSFET, advantages of the proposed p^+^-poly/Al_2_O_3_ MOSFET are its much higher threshold voltage, slightly higher breakdown voltage, and slightly smaller inter-electrode capacitances. Naturally, owing to the elevated threshold voltage, the latter exhibits slightly larger specific on-resistance, high-frequency figure-of-merit, and switching loss. Its threshold voltage has worse temperature stability. Compared with the conventional n^+^-poly/SiO_2_ MOSFET, advantages of the proposed p^+^-poly/Al_2_O_3_ MOSFET are its much lower specific on-resistance, larger threshold voltage at high temperatures, better temperature stability of the threshold voltage, slightly higher breakdown voltage, smaller high-frequency figure of merit and switching loss. Naturally, its threshold voltage at room temperature is smaller, while its inter-electrode capacitances and gate-to-drain charge are larger.

The manufacturing process of the proposed p^+^-poly/high-*k* MOSFET begins with a SiC epitaxial wafer. First, the p-base, n^+^-source, p^+^-contact and JFET regions are sequentially implanted. Aluminum ions for p-type regions should be implanted at high temperatures, and a self-aligned implantation for the n^+^-source region is adopted to form a short channel. The implanted dopants are activated at 1650 °C with the help of a carbon cap.

After stripping the carbon cap and cleaning, a thin SiO_2_ layer is thermally grown and annealed in a NO atmosphere. Then, the high-*k* dielectric is deposited on the SiO_2_ layer using atomic layer deposition technology. Thereafter, the in situ doped p^+^-polysilicon is deposited using a low-pressure chemical vapor deposition process and patterned. The following steps include inter-layer dielectric deposition, source contact etching, Ni deposition and annealing, gate contact etching, metal deposition, passivation layer deposition and pad opening. The last steps are backside wafer thinning and metallization.

## 4. Conclusions

This study demonstrates that the threshold voltage reduction in n-channel 4H-SiC MOSFETs with high-*k* gate dielectrics can be compensated by using a p^+^-polysilicon gate. Because the p^+^-polysilicon has a larger work function than the n^+^-polysilicon, it increases the work-function difference between the polysilicon gate and the surface p-SiC, elevating the threshold voltage of the device by about 1 V compared to the n^+^-polysilicon device. Compared to the conventional SiC MOSFET with a SiO_2_ gate dielectric and an n^+^-polysilicon gate, the proposed SiC MOSFET with a high-*k*/SiO_2_ stacked gate dielectric and a p^+^-polysilicon gate not only achieves a reduction in the specific on-resistance, but also does not sacrifice the threshold voltage. Its threshold voltage has better temperature stability, improving performance at high temperatures. While the breakdown voltage, high-frequency figure of merit and switching loss are improved, the inter-electrode capacitances and gate-to-drain charge increase. This work provides a practical solution for n-channel 4H-SiC MOSFETs adopting high-*k* gate dielectrics.

## Figures and Tables

**Table 1:** Key physical parameters of the dielectrics [17] used in this study.

Material	Dielectric Constant $\varepsilon_{HK}$	Bandgap *E*_g_ (eV)
SiO_2_	3.9	8.9
Al_2_O_3_	9.0	7.0
Y_2_O_3_	15	5.6
HfO_2_	25	5.7

**Table 2:** Key device parameters used in simulations.

Parameter	Unit	Value
Cell pitch	µm	4.8
n^+^/p^+^-polysilicon doping concentration	cm^−3^	1 × 10^20^
Total gate dielectric thickness	nm	40
Thin SiO_2_ thickness	nm	5
Channel length	µm	0.5
Channel doping concentration	cm^−3^	3 × 10^16^
Peak p-base doping concentration	cm^−3^	3 × 10^18^
p-base thickness	µm	1.1
JFET width	µm	1.2
n^−^-drift layer thickness	µm	11
n^−^-drift doping concentration	cm^−3^	1 × 10^16^
n^+^-substrate doping concentration	cm^−3^	1 × 10^19^

**Table 3:** Extracted electrical parameters of the three devices.

Parameters	Unit	n^+^-Poly/SiO_2_ MOSFET	n^+^-Poly/Al_2_O_3_ MOSFET	p^+^-Poly/Al_2_O_3_ MOSFET
*V*_TH_ @ 25 °C	V	2.93	1.83	2.91
*V*_TH_ @ 175 °C	V	2.01	1.31	2.29
*dV*_TH_/*dT*	mV/°C	−6.2	−3.5	−4.1
BV	V	1569	1587	1593
*R*_ON_	mΩ	1670	1200	1220
*R*_ON, SP_	mΩ·cm^2^	3.34	2.40	2.44
*C*_iss_	pF	61.7	95.8	93.3
*C*_oss_	pF	1.8	1.8	1.8
*C*_rss_ (*C*_GD_)	fF	84.5	90.7	90.2
*Q*_GD_	nC	0.42	0.46	0.46
*R*_ON_∗*Q*_GD_	mΩ∗nC	700	556	567
*E*_SW_	µJ	20.7	18.2	18.8

## Data Availability

The data presented in this study are available within the article.
